# Strong Antimicrobial Activity of Silver Nanoparticles Obtained by the Green Synthesis in *Viridibacillus sp*. Extracts

**DOI:** 10.3389/fmicb.2022.820048

**Published:** 2022-02-16

**Authors:** Priyanka Singh, Ivan Mijakovic

**Affiliations:** ^1^The Novo Nordisk Foundation, Center for Biosustainability, Technical University of Denmark, Kongens Lyngby, Denmark; ^2^Systems and Synthetic Biology Division, Department of Biology and Biological Engineering, Chalmers University of Technology, Gothenburg, Sweden

**Keywords:** silver nanoparticles, green synthesis, strong antimicrobial activity, highly stable AgNPs, Gram-negative pathogenic microorganisms, environmental isolate

## Abstract

Recently, green silver nanoparticles (G-AgNPs) have gained much attention in medical science due to their extraordinary effects against multidrug-resistant microorganisms. The strong antimicrobial nature of G-AgNPs corresponds to their unique physicochemical properties such as size, shape, surface charge, and active surface groups available to interact with the pathogens. The current study demonstrates a simple, environmentally friendly, and economical method to produce G-AgNPs from an environmental isolate of *Viridibacillus* sp. The produced G-AgNPs were characterized by various analytical methods, including UV-Vis spectroscopy, single-particle inductively coupled plasma-mass spectrometry (sp-ICP-MS), scanning electron microscopy (SEM), energy dispersive x-ray spectroscopy (EDX), elemental mapping, transmission electron microscopy (TEM), dynamic light scattering (DLS), Fourier-transform infrared spectroscopy (FTIR), and Thermogravimetric analysis (TGA). The reduction of Ag^+^ to Ag° was observed by UV-Vis spectroscopy, which demonstrated the formation of stable G-AgNPs with a Surface Plasmon Resonance (SPR) band at the maximum of 430 nm. TEM analysis demonstrated that the G-AgNPs were spherical with a 5–30 nm size range. The produced G-AgNPs were stable for more than 1 year in an aqueous solution at 4°C. Importantly, G-AgNPs showed remarkable antimicrobial activity against Gram-negative pathogens- *E. coli* and *P. aeruginosa* with MIC values of 0.1 and 4 μg/mL and MBC values of 1 and 8 μg/mL, respectively. This level of antimicrobial activity is superior to other AgNPs reported in the literature.

## Introduction

Silver nanoparticles (AgNPs) are widely known for their industrial applications in the field of medicine, pharmacology, food, agriculture, cosmetics, and textiles due to their unique antimicrobial properties, which further depend on nanoparticles (NPs) structure (Gherasim et al., 2020). The most common methods applied for AgNPs production are physiochemical methods such as laser irradiation, thermal decomposition, electrochemical synthesis, chemical reduction, etc. (Zhang et al., 2018). However, these methodologies also bring many limitations, for instance, the use of toxic materials and volatiles organic solvents, demand for high energy consumption by using high temperature and pressure, the release of harmful byproducts, and toxic waste, which causes potential environmental damage (Garg et al., 2020). The most important limitation is the absorbance of unwanted toxic materials on the surface of produced nanoparticles, which further provide human and environmental toxicity, thus limiting the clinical use of NPs. These limitations motivate the development of green alternative methodologies, which leads to the formation of uniform and stable NPs with a biocompatible layer (called the corona) around them (Singh et al., 2016a; Heinemann et al., 2021). These green nanoparticles have a range of unlimited pharmaceutical applications, including drugs delivery, gene delivery, as a sensor for pathogens detection, and tissue engineering. Various green approaches to produce nanoparticles by using living entities have been reported, such as plant extracts, fungi, yeast, actinomycetes, algae, bacteria, and viruses (Zhang et al., 2020). One such popular approach is using bacteria as a cell factory to produce the AgNPs extracellularly. In addition, to producing biocompatible NPs, bacteria-mediated synthesis is low-cost, environmentally friendly, safe, and simple (Xu et al., 2020; Ssekatawa et al., 2021).

**FIGURE 1:**
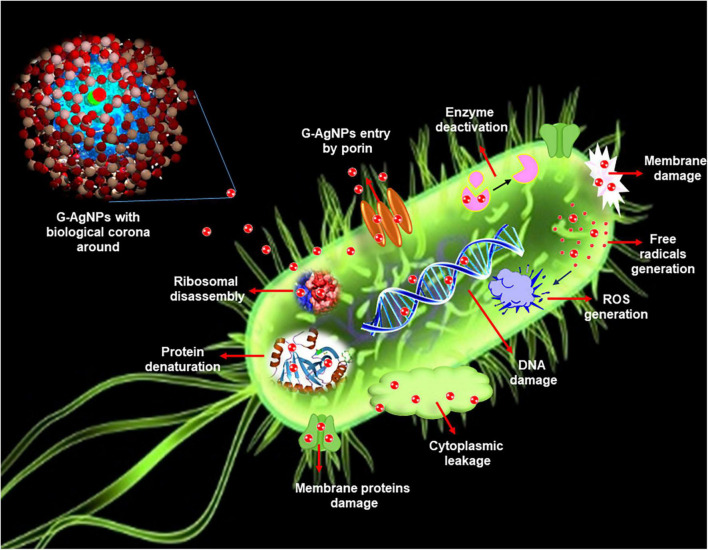
Schematic representation of silver nanoparticles (AgNPs) antimicrobial mechanisms in *Escherichia coli*.

Developing resistance mechanisms in pathogenic microorganisms against current and developing drugs has become a prime concern in the medical field. Understanding the developing resistance mechanisms in these pathogens is important, and designing novel and strong antimicrobial agents that can overcome or circumvent the resistance is equally important (Liu et al., 2021). Indeed, with exposure to novel antimicrobial agents, there are always opportunities for microbes to become unresponsive or resistant. Pathogenic bacteria exert resistance by four different mechanisms: (a) by modification of target proteins, (b) enzymatic degradation or inactivation of drug, (c) decreased membrane permeability which blocks drugs intake, and (d) increased efflux of the drug (Kumar et al., 2021). In this context, G-AgNPs display a broad spectrum of antimicrobial activities and are therefore likely to escape the common mechanisms of resistance development (Prasher et al., 2018). G-AgNPs have been reported as effective treatments against many drug-resistant microorganisms, individually or with traditional/modern antibiotics (Burdusel et al., 2018; Deshmukh S.P. et al., 2019). AgNPs exert killing against multidrug-resistance bacteria by various mechanisms, including membrane damage/leakage, DNA damage, ROS generation, inactivation of intracellular proteins/enzymes, etc. (Figure 1). Recently Ssekatawa et al. (2021) showed the green synthesis of AgNPs from the extract of medicinal plants–*Camellia sinensis* and *Prunus Africana*. The current study deals with the extracellular synthesis of G-AgNPs from an environmental isolate without any additives for the reduction or stabilization process. The extracellular constituents of cells act as reducing and stabilizing agents. In addition to extensive characterization, we explored the synthesized G-AgNPs against *E. coli*, and *P. aeruginosa*, to study their antibacterial property.

## Materials and Methods

### Materials

Silver nitrate (AgNO_3_), tryptic soya agar (TSA), tryptic soya broth (TSB), and Luria broth (LB) were purchased from Sigma-Aldrich Chemicals, St. Louis, MO, United States.

### Identification of Potential Strain

A soil sample was collected in sterile poly bags from the Technical University of Denmark (DTU) field, Lyngby, Denmark. Single colonies were isolated by using the serial dilution technique on TSA plates. All the isolates were tested for primary AgNPs production, and the strongest strain was chosen for further studies. Molecular identification of the isolated potential strain was performed using 16S rDNA amplification and sequencing. Genomic DNA was isolated using the DNeasy Blood and Tissue Kit (Qiagen) and used as a template for PCR with the universal primers 27 F (5′-AGAGTTTGATCMTGGCTCAG-3′) and 1492 R (3′-TACGGYTACCTTGTTACGACTT-5′) (Singh et al., 2017). Eurofins Genomics (Ebensburg, Germany) sequenced the PCR product, and the sequence was analyzed using the NCBI BLAST homepage against the reference sequence database.

### Green Synthesis of Green Silver Nanoparticles

The isolated strain was cultured overnight in 100 mL of TSB, at 37°C, 120 rpm. Next, the growth medium was centrifuged to separate the cells at 8,000 rpm for 10 min. The cell-free supernatant was supplemented with 1 mM AgNO_3_ and incubated in a shake flask incubator at 37°C, 200 rpm, and 24–48 h. The silver salt mix supernatant (reaction medium) was monitored continuously for AgNPs production, by visual inspection, and by recording the UV-Vis spectra of the reaction medium at definite time intervals. Once the G-AgNPs were formed, for purification, the reaction medium was centrifuged at 3,000 for 5 min to remove any big and unwanted components. Then the same medium was centrifuged at 14,000 rpm for 15 min (Singh et al., 2016b). The supernatant was decanted off to collect the pellets then washed several times with distilled water. This residue was suspended again into sterile water and used for all experiments.

### Analytical Characterization of Green Silver Nanoparticles

#### UV-Vis Study

The reduction of silver ions (Ag +) to G-AgNPs was initially monitored *via* visible inspection and then by scanning the reaction medium in UV-Vis spectroscopy at a specific interval. The UV-Vis spectrum was obtained using 6705 UV-Vis spectrophotometer, JENWAY, by scanning 1 mL of the reaction medium in the range of 300–700 nm. The optimization studies for G-AgNPs production were also conducted using visible and UV-Vis spectrum analysis.

#### Single-Particle Inductively Coupled Plasma-Mass Spectrometry

To know the concentration of produced G-AgNPs, sp-ICP-MS (NexION 350D; PerkinElmer Inc., Waltham, MA, United States) was performed. The stability of G-AgNPs was examined by using the purified G-AgNPs and keeping them for a different time, temperatures, and indifferent bacteriological media such as TSB and LB. The results were observed by visible inspection of which pictures are taken, UV-Vis, and sp-ICP-MS analysis before and after the defined period (Singh et al., 2021).

#### Thermogravimetric Analysis Examination

Thermogravimetric analysis (TA Instruments, New Castle, DE, United States) was performed to check the temperature stability G-AgNPs. For analysis, G-AgNPs samples in dried and powdered form were placed in an alumina pan and heated from 20 to 700°C at a ramping time of 10°C/min.

#### Scanning Electron Microscopy

Scanning electron microscopy examination with energy dispersive X-ray (EDX) and elemental mapping was performed to study the G-AgNPs morphology and elemental composition. EDX analysis setup was coupled with the SEM instrument. Sample preparation was done by dropping 5 μl of pure G-AgNPs (0.1 mg/mL) on carbon tape and air-dried at room temperature (RT) for 15 min. SEM Micrographs were recorded using a Quanta FEG 200 ESEM microscope (Quorum Technologies, Hitachi High-Tech Europe GmbH, Sweden).

#### Transmission Electron Microscopy

Transmission electron microscopy study using FEI Tecnai T20 G2 was conducted to analyze the internal morphology, composition, and crystallographic information of G-AgNPs. The instrument was operated at an acceleration voltage of 200 kV. A sample of G-AgNPs was prepared by spotting a drop of pure NPs solution suspended in water on a carbon-coated copper grid. The sample-containing grid was completely dried before analysis.

#### Atomic Force Microscopy

Atomic force microscopy (Park NX20)^1^ measurements were carried out in intermittent contact mode using standard probes of single-crystal highly doped silicon with a radius of curvature of less than 30 nm (SuperSharpSiliconTM Non-contact AFM probes from Nanosensors). The standard uncertainty u(d) of the measured diameters is u(d) < 0.05 day (Singh et al., 2018c).

#### Dynamic Light Sattering Analysis

Dynamic light sattering measurements were performed to study the size distribution concerning intensity and zeta potential of pure G-AgNPs. Particle size measurement was executed using Zetasizer Nano ZS, Chuo-ku Kobe-shi, Japan. The autocorrelation functions of the samples were analyzed using the Contin algorithm through the Zetasizer 7.12 software. Samples were run in triplicates (Wypij et al., 2021).

#### Fourier Transform-Infrared Spectroscopy

Green silver nanoparticles were subjected to Fourier transform infrared (FTIR) analysis to determine the presence of biomolecules, functional groups responsible for the reduction and capping/stabilization. The FTIR measurements were carried out using Nicolet iS50 (Thermo Fisher Scientific, Waltham, MA, United States) by scanning the air-dried purified G-AgNPs and freeze-dried cell’s supernatant within the range of 500–4,000 cm^–1^. The recorded spectra recorded were plotted as transmittance (%) vs. wavenumber (cm^–1^).

### Antimicrobial Activity of Green Silver Nanoparticles

#### Green Silver Nanoparticles Effects on Gram-Negative Pathogens

The antimicrobial activity of G-AgNPs was evaluated against two Gram-negative pathogens: *Escherichia coli* UTI 89, and *Pseudomonas aeruginosa* PAO1. Both the strains were grown overnight in LB medium at 37°C for 24 h. The overnight grown cultures were diluted to approximately 1–2 × 10^5^ colony-forming units (CFU)/mL using LB medium. Then, the G-AgNPs were added in concentrations ranging from 0.1 to 16 μg/mL. The LB medium containing respective pathogenic bacteria and G-AgNPs were further incubated in a shake flask incubator at 37°C, 150 rpm, for 24 h. After 24 h, the samples were analyzed by measuring the optical density (OD) at 550 nm. The MIC was defined as the lowest concentration of G-AgNPs, which inhibited the bacterial growth, measured as OD_550_. The MBC value was defined as the lowest concentration of G-AgNPs required to kill the respective bacterial strain. To measure MBC, 100 μL of the LB medium containing respective pathogenic bacteria and G-AgNPs were spread on agar plates and incubated at 37°C overnight, followed by a CFU count.

#### Live and Dead Staining

To visualize the viable and dead cells, control cells and cells treated with G-AgNPs were stained for 20 min with a mixture of 6.0 μM SYTO 9 and 30 μM KI from Live/Dead BacLight Viability kit L13152 (Invitrogen, Molecular Probes, Inc., Eugene, OR, United States). Fluorescence microscopic imaging of the cells was performed using a LEICA DM 4000 B (Leica Microsystems, Copenhagen, Denmark).

#### Scanning Electron Microscopy Analysis of Treated Cells

To evaluate the drastic effects of G-AgNPs on individual cells, SEM was carried out. SEM was performed by fixing the control and treated cells with 3% of glutaraldehyde overnight at 4°C. The next day, the samples were dehydrated with graded series of ethanol concentrations (40, 50, 60, 70, 80, and 90%) for 15 min and with absolute ethanol for 20 min. The dehydrated samples were placed on SEM carbon tape and left to dry at RT. The samples were coated with gold before SEM imaging. EDX and elemental mapping of G-AgNPs treated cells was also performed to check that the killing effects are due to the action of G-AgNPs only.

## Results

### Molecular Characterization of the Isolate

The rRNA sequencing of isolated bacterial strain indicated 99.05% identity with *Viridibacillus arvi* strain LMG 22165. The isolated strain sequence number is submitted to NCBI with GenBank ID: SUB10641455. *Viridibacillus arvi* is reported to be Gram-positive, aerobic, spore-forming, rod-shaped bacteria (Albert et al., 2007). Based on 16rRNA sequence similarity, the isolated strain was referred to as *Viridibacillus* sp.

### Green Synthesis of Green Silver Nanoparticles

**FIGURE 2:**
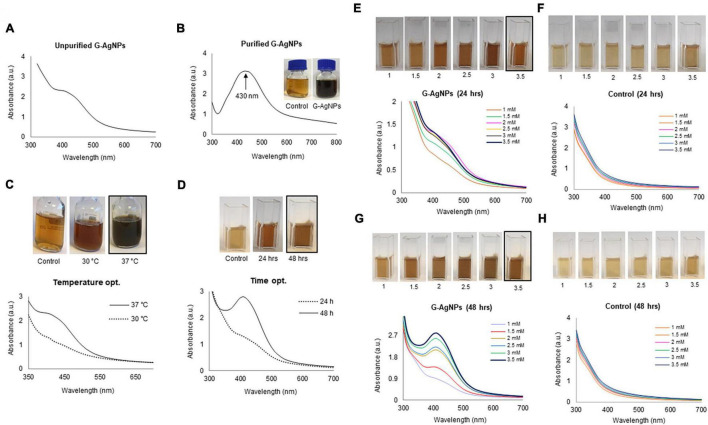
Visible and UV-Vis spectral examination of optimization studies for green silver nanoparticles (G-AgNPs) production. **(A)** UV-Vis peak of unpurified G-AgNPs after 48 h of synthesis, **(B)**, purified G-AgNPs. **(C)** Visible and UV-Vis peaks for temperature optimization, **(D)** time optimization, **(E–H)** salt optimization for G-AgNPs production at 24 and 48 h.

The supernatant of a culture of *Viridibacillius* sp. grown for 24 h was used as a reaction medium to reduce silver salt and provide capping/stabilizing components to the formed G-AgNPs. The culture supernatant was supplemented with 1 mM AgNO_3_ and incubated further in a shake flask incubator at 37°C, 200 rpm, for 24–48 h. After the incubation period, the supernatant showed a visible color change from pale yellow to deep brown, which is attributed to the surface plasmon resonance (SPR) property of G-AgNPs (Ronavari et al., 2021). The observation was further confirmed by simultaneously recording the SPR band *via* UV-Vis at a specified time interval. After incubation, the reaction medium was first directly scanned, and then the purified G-AgNPs samples were also scanned to confirm the peaks intensity and overlapping range (Figures 2A,B). Purified G-AgNPs showed a strong and sharp SPR peak at 430 nm. The kinetics of G-AgNPs formation was monitored by recording the spectrum of the reaction medium at different temperatures, times, and various salt concentrations. For temperature optimization studies, the highest and clear peak in UV-Vis spectra was observed at 37°C (Figure 2C). According to the time-resolved UV-Vis spectra, the SPR absorbance band increased with the reaction time for up to 48 h (Figure 2D). For the salt concentration optimization, as the salt concentration increased, the intensity of the SPR band also increased. This trend continued to up to 3.5 mM (Figures 2E–H); any further increase in salt concentration led to broadening the peak and accumulation of NPs in the reaction medium. Thus the optimal conditions for G-AgNPs production using cell-free supernatant of *Viridibacillus* sp., were AgNO_3_ concentration of 3.5 mM, the temperature of 37°C, and the incubation period of 48 h. Any deviations from these key parameters resulted in disturbance of the UV-Vis peaks, signifying particle agglomeration.

### Characterization of Green Silver Nanoparticles

**FIGURE 3:**
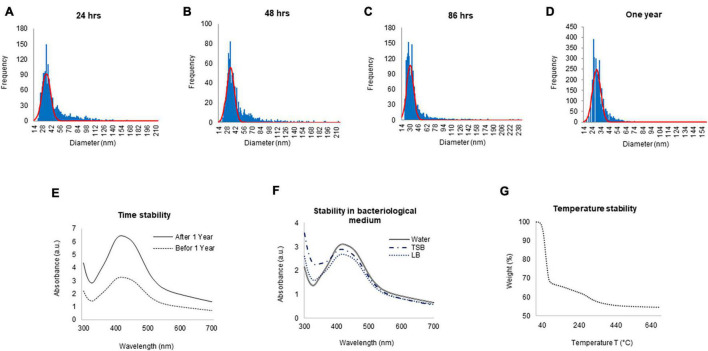
ICPMS and stability analysis of green silver nanoparticles (G-AgNPs). ICPMS histogram of G-AgNPs at different time intervals **(A)** 24 h, **(B)** 48 h, **(C)** 86 h, **(D)** 1 year. UV-Vis spectrum representing the stability analysis of G-AgNPs **(E)** before and after 1 year, **(F)** in a different medium, **(G)** at the temperature range from 20 to 700°C measured by the TGA instrument.

To check the yield of G-AgNPs, sp-ICP-MS was used. The result showed that the measured total mass concentration of G-AgNPs was 0.078 μg/μl, with a negligible dissolved fraction (<0.1 ppb). We then examined the stability of G-AgNPs over short and long periods. The measurements were made after 24, 48, 86 h, and 1 year (Figures 3A–D). Our study found no significant differences in particle size recorded using sp-ICP-MS, resulting in mean diameter of 15–60 nm, thus indicating the long-term stability of produced G-AgNPs. Based on visible observation, no dissolution or agglomeration of G-AgNPs occurred even after 1 year of storage in an aqueous solution. In addition, UV-Vis observations of G-AgNPs showed a sharp and overlapping peak before and after 1 year, thus confirming their aqueous stability (Figure 3E). A bacterial growth medium, such as TSB and LB, and water were tested for G-AgNPs stability (Figure 3F). The results showed that the G-AgNPs are completely stable in water as well as in the growth media. For thermal stability, the TGA measurement was taken (Figure 3G). According to the obtained spectra, there were two stages of weight loss, first at 150°C and second at 400°C. The physical adsorption of water molecules caused the initial loss of weight up to 150°C on the surfaces of the G-AgNPs. At 400°C, most of the weight loss was due to the biomolecules decomposing and evaporating from the surface of the G-AgNPs (Deshmukh A.R. et al., 2019). A further increase in temperature led to the complete degradation of G-AgNPs.

**FIGURE 4:**
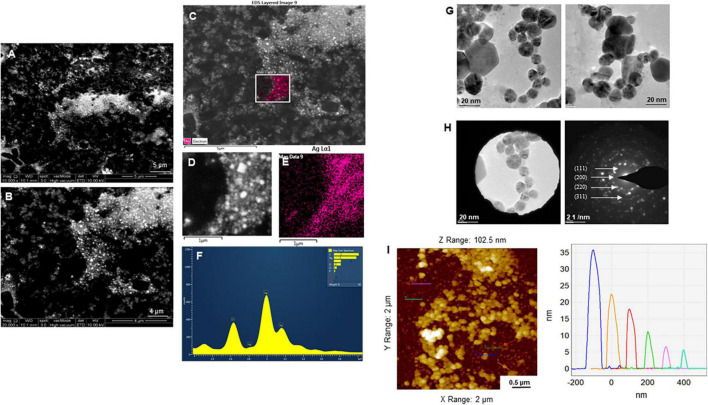
Structural analysis of green silver nanoparticles (G-AgNPs). **(A,B)** Scanning electron microscopy (SEM) images of nanoparticles, **(C–E)** elemental mapping of G-AgNPs showing scanned image of NPs and respective region with the individual silver nanoparticles (pink color), **(F)** energy dispersive x-ray spectroscopy (EDX) spectrum of the elemental mapped region is showing highest peak for silver element. **(G)** Transmission electron microscopy (TEM) image of G-AgNPs showing spherical nanoparticles, **(H)** selected area (electron) diffraction (SAED) pattern of G-AgNPs, and **(I)** AFM size analysis representation of G-AgNPs.

**FIGURE 5:**
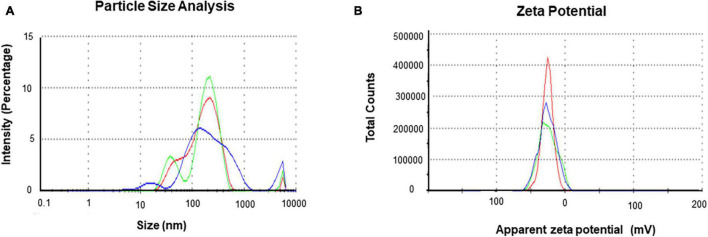
Dynamic light scattering (DLS) analysis of green silver nanoparticles (G-AgNPs) **(A)** nanoparticles distribution concerning size and intensity and **(B)** zeta potential of G-AgNPs representing highly negative surface charge.

The purity and crystalline nature of G-AgNPs that were produced under optimized conditions were investigated by conducting SEM, EDX, elemental mapping, TEM, and Selected area (electron) diffraction (SAED) studies. SEM image examination showed the spherical structure of G-AgNPs (Figures 4A,B). The elemental mapping results demonstrated the selected scanned area of the G-AgNPs sample (Figures 4C–E) resembles the silver element (pink color) (Deshmukh A.R. et al., 2019). The elemental composition of G-AgNPs was determined *via* EDX spectroscopy, which reveals the presence of the strong elemental signal from silver at 3 keV (Figure 4F; Singh et al., 2018a). The core size and morphology of G-AgNPs were determined *via* TEM, which displayed the G-AgNPs are approximately spherical and uniformly distributed with an average particles size of 5–30 nm (Figure 4G). However, few polydispersity were found in hexagonal and truncated triangular form NPs. This morphology of nanoparticles depends on the experimental conditions and the constituents of the cellular supernatant. The SAED pattern of G-AgNPs indicated characteristic rings at 111, 200, 220, and 311 crystallographic planes, which corresponds to the face-centered cubic (fcc) of AgNPs (Figure 4H). These values are in accord with those reported in earlier studies (Sangaonkar and Pawar, 2018); they suggest the crystalline nature of AgNPs. Moreover, the AFM analysis also revealed similar size distribution, from 5 to 35 nm (Figure 4I). Hydrodynamic diameter and surface charge of the produced G-AgNPs were determined by DLS. The diameter and PDI were found to be 154.4 nm and 0.378, respectively (Figure 5A). The zeta potential value of the aqueous G-AgNPs solution at RT was found to be −25.7 mV (Figure 5B), which suggested that the G-AgNPs are negatively charged and quite stable at neutral conditions.

**FIGURE 6:**
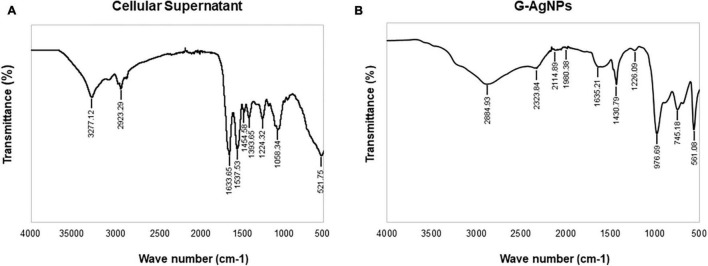
Fourier-transform infrared spectroscopy (FTIR) spectrum of panel **(A)** freeze-dried cellular supernatant and **(B)** green silver nanoparticles (G-AgNPs), which demonstrate the active surface groups for respective samples.

Fourier-transform infrared spectroscopy measurements were conducted to identify the extracellular components released from isolated strain, present in the reaction medium, responsible for reducing, capping, and stabilizing G-AgNPs. The FTIR spectra of freeze-dried cell-free supernatant and purified G-AgNPs are shown in Figures 6A,B and Table 1. Comparing the FTIR spectrum of cellular supernatant with G-AgNPs, the high broad peaks for G-AgNPs appear at 2884.93 (asymmetric and symmetric C-H stretching, or secondary amines), 1635.21 [carboxyl groups (-C=O), and carbonyl group (-C=O)-stretching vibration of proteins], 1430.79 (C-H bending of COO^–^ or carboxylate groups), 561.08 which are identical to the supernatant spectrum. The FTIR results indicate the presence of carboxyl groups (-C=O), and amine groups (−NH) which represents the presence of proteins, amino acids, and other biomolecules originating from the supernatant on the surface of the produced G-AgNPs, responsible for capping and stabilizing G-AgNPs (Abbai et al., 2016). The FTIR spectrum proved that the reaction medium contains reducing and stabilizing agents such as sugar, proteins, and amino acids responsible for the green synthesis of G-AgNPs.

**TABLE 1:** Fourier transform-infrared spectroscopy (FT-IR) spectra of cellular extract and green silver nanoparticles (G-AgNPs).

Type of Bond	Cellular extract Wavenumber (cm^–1^)	G-AgNPs Wavenumber (cm^–1^)
-OH (hydroxyl group) of phenolic compounds and N-H group	3277.12	
asymmetric stretching of a methyl group -CH_3_ C-H stretching of alkanes or secondary amine	2923.29	2884.93
Alkyne group		2114.89, 1980.38
-C=O stretching vibration in flavonoids and terpenoids, and carbonyl group (-C=O) stretching vibration of proteins or amide I)	1633.65, 1537.53	1635.21
N-H stretching vibration of proteins	1454.58	1430.79
C-N aromatic amino groups	1393.65	
Overlapping of C-O, C-N, C-O-C and C-O-P stretching modes	1058.34	
C-C deformation	521.75	561.08

### Strong Antimicrobial Activity of Green Silver Nanoparticles

**FIGURE 7:**
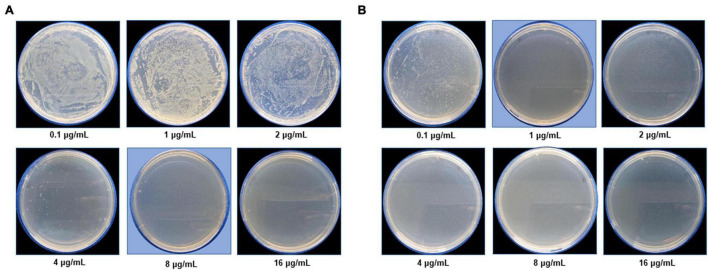
Cell viability test at a different concentration range from 0.1 to 16 μg/mL for **(A)** *Escherichia coli* and **(B)** *Pseudomonas aeruginosa* after green silver nanoparticles (G-AgNPs) treatment. The blue background shows the MBC values of G-AgNPs for respective pathogens.

**FIGURE 8:**
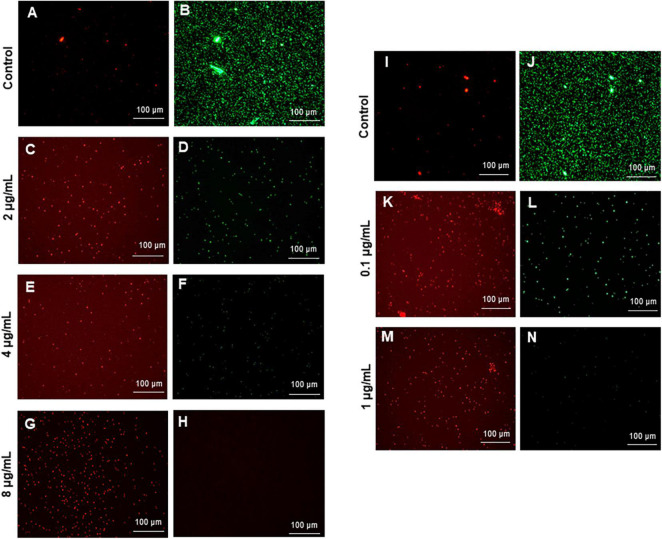
Live and dead staining of **(A–H)** *Escherichia coli*, and (I-N) *Pseudomonas aeruginosa* after treatment with green silver nanoparticles (G-AgNPs) at selected concentrations. *E. coli* cells: **(A,B)** control without G-AgNPs; **(C,D)** 2 μg/mL; **(E,F)** 4 μg/mL; **(G,H)** 8 μg/mL of G-AgNPs. *P. aeruginosa* cells: **(I,J)** control without G-AgNPs; **(K,L)** 0.1 μg/mL; **(M,N)** 1 μg/mL of G-AgNPs.

**FIGURE 9:**
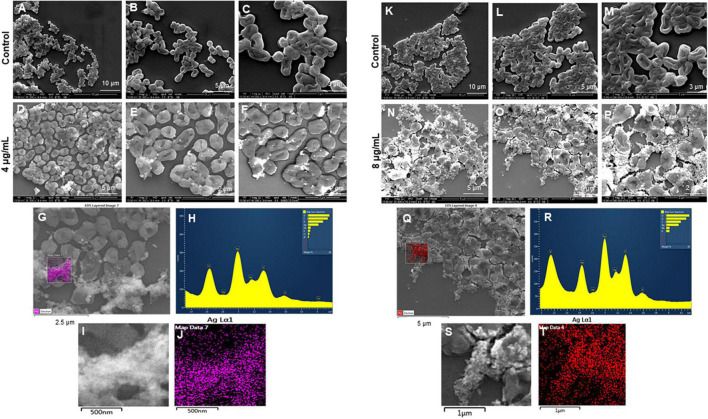
Scanning electron microscopy (SEM) analysis of *Escherichia coli* cells after treatment with green silver nanoparticles (G-AgNPs). **(A–F)** Control *E. coli* cells and G-AgNPs treated 4 μg/mL at different scales. **(G)** Scanned image of treated cells **(H)** energy dispersive x-ray spectroscopy (EDX) spectrum of choose area **(I,J)** elemental mapping of the selected area showing silver element in the treated cells, **(K–P)** control *E. coli* cells and G-AgNPs treated cells with 8 μg/mL at different scales. **(Q)** Scanned image of treated cells **(R)** energy dispersive x-ray spectroscopy (EDX) spectrum of choose area **(S,T)** elemental mapping of the selected area showing silver element in the treated cells.

**FIGURE 10:**
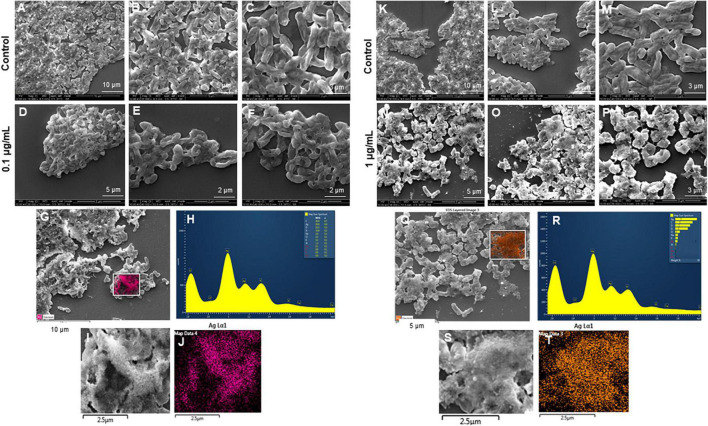
Scanning electron microscopy (SEM) analysis of *Pseudomonas aeruginosa* cells after treatment with green silver nanoparticles (G-AgNPs). **(A–F)** Control *P. aeruginosa* cells and G-AgNPs treated cells with 0.1 μg/mL at different scales. **(G)** Scanned image of treated cells **(H)** energy dispersive x-ray spectroscopy (EDX) spectrum of choose area **(I,J)** elemental mapping of the selected area showing silver element in the treated cells, **(K–P)** control *P. aeruginosa* cells and G-AgNPs treated cells with 1 μg/mL at different scales. **(Q)** Scanned image of treated cells **(R)** energy dispersive x-ray spectroscopy (EDX) spectrum of choose area **(S,T)** elemental mapping of the selected area showing silver element in the treated cells.

The most remarkable feature of the G-AgNPs synthesized in this study is their strong bacteriostatic and bactericidal activity against pathogenic *E. coli* and *P. aeruginosa*. The recorded MIC values against *E. coli* and *P. aeruginosa* were 4 and 0.1 μg/mL, respectively, while the respective MBC values were 8 and 1 μg/mL (Figures 7A,B). We used the live and dead staining technique to confirm the viability results. This technique allows one to distinguish the live cells (stained green) and dead cells (stained red) under a fluorescence microscope (Figures 8A–N). These results confirmed a dramatic onset of killing bacterial cells at G-AgNPs concentrations above 4 μg/mL for *E. coli* (Figure 8A–H) and 0.1 μg/mL for *P. aeruginosa* (Figures 8I–N). To investigate whether the killing effects involve drastic morphological changes in treated cells, we used SEM. A significant morphological alteration was observed in G-AgNPs treated cells *E. coli* cells (Figures 9A–F,K–P) and *P. aeruginosa* cells (Figures 10A–F,K–P). The severity of these effects was correlated to the concentration of applied G-AgNPs for both bacterial species. To confirm the damage that G-AgNPs provoked, we performed EDX and elemental mapping of individual cells. The results disclosed that the damaged cells generate a clear peak of the silver element in the EDX spectrum for *E. coli* (Figures 9G–J,Q–T) and *P. aeruginosa* cells (Figures 10G–J,Q–T). In addition, the mapping results also resemble the silver element in the scanned image of treated cells, indicating that G-AgNPs get internalized.

## Discussion

The cell-free supernatant of environmental isolate *Viridibacillus* sp. acted as a reducing and stabilizing agent, which led to the formation of highly stable and monodisperse G-AgNPs. The reduction process does not require any additional reducing or stabilizing agents. Moreover, the synthesis took place in the cell-free medium, which means with the help of extracellularly released biomolecules from the isolated *Viridibacillus* sp. The extracellular components in the reaction medium form a biological corona around the nanoparticles, which helps long-term stabilization. This is an important feature of bacteria-mediated extracellular synthesis. Unlike intracellular synthesis, extracellular synthesis provides an opportunity to avoid additional steps in downstream processing, such as cell disruption by membrane lysis or sonication, removal of insoluble components, extraction of complete nanoparticles from intracellular organelles, etc. (Singh et al., 2018b). Thus, the proposed methodology is more economical once an appropriate bacterial strain is identified (Kapoor et al., 2021).

The G-AgNPs formation in our study was supported by visual observation and UV-Vis analysis. Based on kinetics and optimization studies, no significant change in the absorbance was observed beyond the optimized parameters, which suggested that the nucleation and growth process during this period supported the complete reduction of silver salt into G-AgNPs. Moreover, the color, ICPMS, and UV-Vis spectrum of G-AgNPs remained stable for more than 1 year and showed no aggregation (Singh et al., 2021). FTIR was used as a powerful tool to study the functional molecular vibrations. The spectrum of freeze-dried cell-free supernatant of *Viridibacillus* sp. showed that the medium contains proteins, reducing sugars, polysaccharides, various biomolecules, and amino acids, which help reduce and inhibit further agglomeration of produced G-AgNPs. This is the most important advantage of using green nano factories to produce AgNPs. It provides the additional biocompatible layer, which can keep nanoparticles stable for many years without any additives, thus enhancing colloidal stability (Belteky et al., 2019). In contrast, the physically or chemically produced NPs lack the additional biocompatible layer and require a surplus stabilizer. Most of the NPs produced by physical or chemical methods show complete agglomeration with time, even in the presence of stabilizing agents (Deshmukh A.R. et al., 2019).

Silver nanoparticles effects on pathogenic bacteria are well known (Figure 1; Liao et al., 2019; Crisan et al., 2021). The effects of AgNPs on Gram-negative and Gram-positive bacteria are mainly influenced by the thickness and composition of the cell wall, which means that Gram-negative bacteria are more susceptible, and Gram-positive bacteria can show resistance to some extent. Except for the thin cell membrane, the lipopolysaccharides (LPS) in the cell membrane promote the chemical interaction of the membrane with AgNPs (Loo et al., 2018). Although many studies have reported the antimicrobial activity and possible action mechanism of biological AgNPs, strong effects at a very low concentration of AgNPs are rare. The produced G-AgNPs were explored against two Gram-negative strains in the current study. Results showed extremely strong antimicrobial activity, i.e., total killing at 8 μg/mL for *E. coli* and 1 μg/mL for *P. aeruginosa*. The mentioned concentrations for total killing are very low compared to other reported green AgNPs. For instance, recently, Shah et al. (2021) showed that the LD50 dose (concentration of AgNPs causing 50% inhibition) obtained from *Plantago lanceolate* against *E. coli* was 45.54 mg/L, which is much higher than the G-AgNPs MBC value (kill 100% bacteria) against *E. coli*, i.e., 8 μg/mL, in our study. Similarly, Devanesan and AlSalhi, 2021 demonstrated the antimicrobial activity of AgNPs originated from flower extract of *Abelmoschus esculentus*. The authors showed the MBC value of AgNPs against *E. coli* and *P. aeruginosa* were 110 and 105 μg/mL (Devanesan and AlSalhi, 2021). Loo et al. (2018) described the MBC value of extremely small 4 nm AgNPs produced using pu-erh tea leaves against *E. coli* as 7.8 μg/mL. Ssekatawa et al. (2021) described the MBC value of two green AgNPs originating from *Prunus africana* and *Camellia sinensis*. The MIC and MBC value of 125 and 250 μg/mL against *E. coli* (Ssekatawa et al., 2021). Thus, our G-AgNPs showed MBC at 8 and 1 μg/mL against *E. coli* and *P. aeruginosa*, superior to all the cited examples. The possible reason for the strong antibacterial ability of G-AgNPs could be the biological corona, which provides a high negative surface charge, spherical shape of NPs, which allow them to interact with pathogens with the maximum surface area available.

We further confirmed the cells’ death by SEM. SEM has revealed that after contact with G-AgNPs, the cell membrane of *E. coli* and *P. aeruginosa* cells is completely ruptured. Thus, the G-AgNPs attach onto the negatively charged surface of the cell wall and membrane, which leads to the shrinkage of the cytoplasm and membrane detachment, finally leading to rupture of the cell wall. In addition, the interaction of G-AgNPs with the sulfur-containing proteins present in the cell wall could also affect the membrane permeability and cause cell leakage. It is reported that the porins on Gram-negative bacteria are also responsible for AgNPs uptake. Following penetration, G-AgNPs may interact with cellular components such as proteins, lipids, and DNA, corresponding to the damaging effects (Ullah Khan et al., 2018). AgNPs also cause DNA damage, mutations, inhibition of enzymes and proteins (Singh et al., 2020). It has been found that Ag (+) ions intercalate between the purine and pyrimidine base pairs, disrupt the H-bonds between base pairs of the anti-parallel DNA strands, and thereby disrupt the double-helical structure (Joshi et al., 2020). Another well-known mechanism of AgNPs action is their ability to produce ROS and free radical species and consequent increase in oxidative stress in cells and apoptosis (Loo et al., 2018; Ullah Khan et al., 2018). All these mechanisms were presented in Figure 1.

One of the most important physicochemical properties that affect antimicrobial activity is the size and shape of NPs (Karade et al., 2021). Typically, smaller NPs have the larger surface area available to interact and ascend intracellular penetration (Ginjupalli et al., 2018). We hypothesized that the bigger G-AgNPs >10 nm could cause membrane damage. In contrast, the smaller NPs (less than 10 nm) could enter the cells after adhesion and damage the intracellular structures, thus affecting vital cellular functioning. Thus, we believe that the size range of G-AgNPs from 5 to 30 nm offered strong interaction of nanoparticles on the surface (bigger NPs) and internal organelles (smaller NPs). In addition, sphere-shaped or quasi-spherical AgNPs are more susceptible to releasing Ag + ions; thus, G-AgNPs showed high antimicrobial effects. Moreover, as mentioned above, strong negative zeta potential and biological corona also play an important role in providing strong antimicrobial activity and stability in the biological environment so that NPs won’t degrade and act with their full potential. Thus, we strongly believe that the strong antimicrobial activity of produced G-AgNPs is due to the above-discussed mechanism. Exploring these nanoparticles further against more multidrug-resistant pathogens will answer the desire for strong antimicrobial agents in medical fields.

## Conclusion

Green silver nanoparticles were successfully formed *via* a green synthetic method using cell-free supernatant of *Viridibacillus* sp., which acted as a reducing and capping agent. The G-AgNPs production was confirmed by the appearance of SPR band 430 nm and found to be highly stable, crystalline, and nearly spherical in size. The G-AgNPs showed excellent antimicrobial activity against two Gram-negative strains at very low concentrations. We propose that these nanoparticles constitute a promising opportunity for developing antimicrobial weapons with efficient antimicrobial properties, stability, and recyclability.

## Data Availability Statement

The datasets presented in this study can be found in online repositories. The names of the repository/repositories and accession number(s) can be found in the article/supplementary material.

## Ethics Statement

Ethical review and approval was not required for the study on human participants in accordance with the local legislation and institutional requirements. Written informed consent for participation was not required for this study in accordance with the national legislation and the institutional requirements.

## Author Contributions

PS designed and performed the experiments, analyzed the results, and prepared the manuscript and figures. IM supervised all experimental work and edited the manuscript. Both authors contributed to the article and approved the submitted version.

## Conflict of Interest

The authors declare that the research was conducted in the absence of any commercial or financial relationships that could be construed as a potential conflict of interest.

## Publisher’s Note

All claims expressed in this article are solely those of the authors and do not necessarily represent those of their affiliated organizations, or those of the publisher, the editors and the reviewers. Any product that may be evaluated in this article, or claim that may be made by its manufacturer, is not guaranteed or endorsed by the publisher.

## Footnotes

1
www.parkafm.com
